# KOH-activated biochar derived from *Pinus brutia* wildfire residues for efficient sulfamethoxazole removal: adsorption mechanisms and process optimization

**DOI:** 10.1039/d6ra06348a

**Published:** 2026-08-18

**Authors:** Lütfi Erden

**Affiliations:** a Çanakkale Onsekiz Mart University, Can Vocational School, Department of Electricity and Energy Çanakkale Türkiye lutfi.erden@comu.edu.tr

## Abstract

Sulfamethoxazole (SMX) is a persistent antibiotic frequently detected in aquatic environments, posing significant risks to ecosystem health and promoting antibiotic resistance. In this study, a highly porous KOH-activated biochar (KA-BC-WFR) was synthesized from wildfire residues of *Pinus brutia* and applied for the efficient removal of SMX from aqueous solutions. Textural characterization revealed a highly developed hierarchical pore structure; N_2_ adsorption–desorption isotherms determined a high BET specific surface area of 828 m^2^ g^−1^ with an average pore diameter of 1.30 nm, confirming a predominantly microporous structure, while Scanning Electron Microscopy (SEM) displayed a sponge-like morphology with macroporous transport channels ranging from 1.0 to 9.6 µm. The point of zero charge (pH_PZC_) of the biochar was determined to be 6.24 using the pH drift method. Response Surface Methodology (RSM) optimized the process, identifying pH 2.0 as the optimal condition. Kinetic studies followed the pseudo-second order model, and equilibrium data were best fitted by the Redlich–Peterson and Toth models. The maximum monolayer adsorption capacity (*q*_max_) was 160.05 mg g^−1^ at 298 K. Thermodynamic parameters confirmed that the process was spontaneous (Δ*G*° < 0) and exothermic (Δ*H*° = −11.93 kJ mol^−1^). The adsorption mechanism was attributed to a synergistic effect of rapid pore filling, strong hydrogen bonding between the neutral SMX species and surface functional groups, and π–π stacking interactions. These findings demonstrate that converting wildfire residues into activated biochar is a highly effective strategy for pharmaceutical wastewater remediation.

## Abstract 2

KOH-activated biochar derived from *Pinus brutia* wildfire residues offers a highly efficient, kinetically fast remediation strategy for sulfamethoxazole removal.

## Graphical abstract

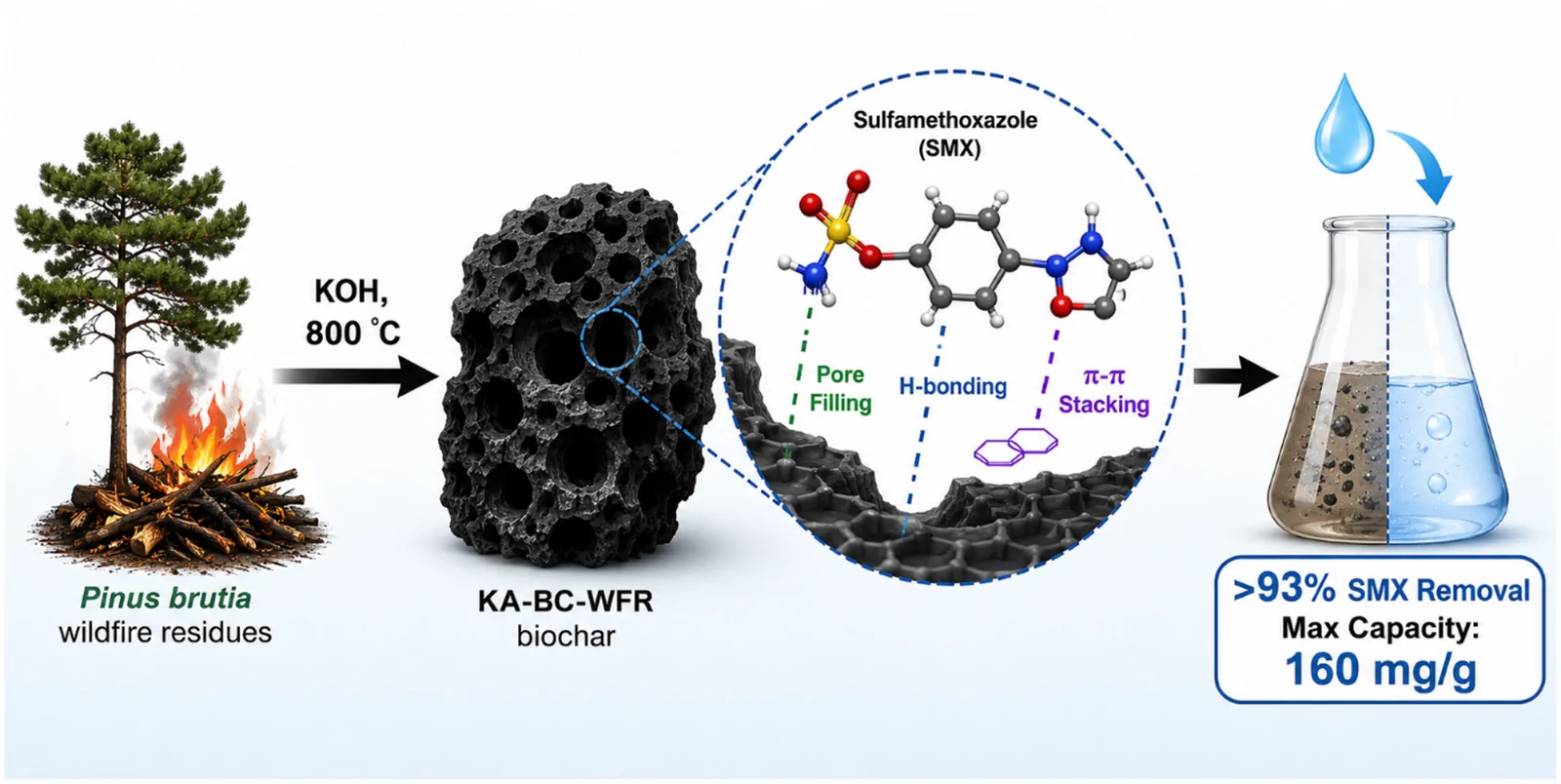

## 1. Introduction

The rapid escalation of pharmaceutical consumption in human and veterinary medicine has led to the emergence of pharmaceutically active compounds (PhACs) as a critical class of environmental contaminants. Among these, antibiotics are of particular concern due to their potential to induce antibiotic resistance in bacteria, posing a severe threat to global public health and aquatic ecosystems.^1–4^ Sulfamethoxazole (SMX), a widely prescribed sulfonamide antibiotic, is frequently detected in surface water, groundwater, and wastewater effluents due to its high solubility, poor biodegradability, and incomplete removal by conventional wastewater treatment plants.^1,5^

Various treatment technologies have been investigated for the remediation of SMX-contaminated waters, including membrane filtration, advanced oxidation processes (AOPs), photocatalysis, and biological treatment.^6–9^ However, many of these methods suffer from significant drawbacks. Biological treatments are often inefficient against persistent antibiotics like SMX and can be inhibited by their toxicity.^9^ Membrane technologies require high energy input and face fouling issues, while AOPs can generate toxic by-products that may be more harmful than the parent compound.^6^ Consequently, there is an urgent need for cost-effective, energy-efficient, and environmentally friendly alternatives.

Adsorption has emerged as one of the most promising techniques for the removal of micropollutants due to its simplicity of design, ease of operation, insensitivity to toxic substances, and high removal efficiency.^10,11^ The key to an effective adsorbent lies in a high surface area, a well-developed porous structure for diffusion, and specific surface functional groups that can interact with the target pollutant. Recently, the integration of advanced interpretative models, machine learning, and comprehensive mechanistic studies has become essential to accurately evaluate the binding behaviors of complex pollutants—such as antibiotics and phosphates—onto engineered and functionalized biochars.^12–14^

In this context, the valorization of lignocellulosic waste into activated biochar presents a practical approach for environmental remediation. The increase in forest fires has generated vast quantities of wildfire residues, particularly from species like *Pinus brutia*. Utilizing air-carbonized wood, which is carbonaceous matter formed from the burning of biomass in air, as a precursor for the production of activated carbon has been demonstrated as a highly viable strategy. Previous research on air-carbonized wood has successfully yielded highly microporous carbons with exceptional packing density and unprecedented performance for CO_2_ capture applications.^15^ Leaving these residues in forests can pose pest risks and fuel future fires; thus, converting this waste into high-value adsorbents aids in forest management while providing a low-cost precursor for water treatment.

This study focuses on the synthesis of KOH-activated biochar from wildfire residues (KA-BC-WFR) and its application for the sequestration of SMX. The primary objective of this work was to rigorously evaluate its performance as an adsorbent using Response Surface Methodology (RSM) to optimize critical process variables. Furthermore, a comprehensive analysis of adsorption kinetics (*via* non-linear regression), isotherms, and thermodynamics was conducted to elucidate the rate-limiting steps and the precise nature of the interaction mechanism.

## 2. Materials and methods

### 2.1. Chemicals and reagents

The pine samples (*Pinus brutia*) that were not completely burned but were carbonized due to the high temperatures of the fire and the excessive CO_2_ gases released during the fire were collected from seven different locations in the Çanakkale forest fire region and mixed. Potassium hydroxide (KOH, ≥85% purity) was used as an activation agent on the samples. The sulfamethoxazole (SMX) was used from Sigma-Aldrich, purity >99%.

### 2.2. Adsorbent synthesis

The precursor material consisted of wildfire residues (*Pinus brutia*) collected from the Çanakkale region. The collected pine samples were first crushed into powder, washed thoroughly with distilled water to remove surface ash and impurities, dried overnight at 110 °C, and then sieved to a particle size of 40–80 mesh (0.420–0.177 mm) to ensure uniform particle dimensions.

The feasibility of utilizing *Pinus brutia* wildfire remnants as a highly effective adsorbent precursor was recently established in our preliminary investigations, which demonstrated that base-activated biochar from this specific waste stream achieved a maximum adsorption capacity of 130.25 mg g^−1^ and a 96.77% removal efficiency for cationic dyes.^16^ Building upon these foundational findings, the specific activation parameters for the present study were selected based on further extensive screening of various activation temperatures (600, 700, and 800 °C) and chemical agents (ZnCl_2_, NaOH, and KOH). These trials demonstrated that KOH activation at 800 °C provided the optimal balance of hierarchical porosity and surface functionalization required for this precursor to efficiently capture SMX.

Consequently, chemical activation was performed by mixing the clean powdered samples with analytical-grade potassium hydroxide (KOH, ≥85% purity) at a 1 : 1 ratio by weight. A specific volume of deionized water (approximately 5 mL per gram of mixture) was added to form a slurry, which was agitated using a magnetic stirrer at 200 rpm for 1 hour to ensure uniform impregnation of the KOH into the biochar matrix.

The dried impregnated mixture was placed in a ceramic boat crucible and inserted into a tube furnace. Activation was carried out under a continuous nitrogen (N_2_) flow of 200 mL min^−1^ with a heating ramp rate of 10 °C min^−1^ up to the target temperature of 800 °C, where it was held for 2 hours. Following activation, the sample was allowed to cool naturally to room temperature under the continuous N_2_ atmosphere to prevent oxidation.

To remove unreacted potassium compounds and residual ash, the resulting material was rigorously washed. The sample was first stirred in a 0.1 M HCl solution (50 mL per gram of biochar) for 2 hours, followed by repeated vacuum filtration and washing with hot deionized water until the filtrate reached a neutral pH (pH ∼ 7.0). Finally, the activated biochar, standardized as KA-BC-WFR, was dried in an oven at 110 °C for 24 hours and stored in a desiccator prior to use.

### 2.3. Characterization

The surface morphology of KA-BC-WFR was visualized using a Scanning Electron Microscope (SEM, JEOL JSM-7100-F). The surface functional groups were identified using Fourier Transform Infrared Spectroscopy (FTIR, PerkinElmer) in the range of 4000–400 cm^−1^. The point of zero charge (pH_PZC_) of the adsorbent was determined utilizing the pH drift method to evaluate the surface charge behavior. All FTIR measurements were performed in triplicate on independent samples to ensure the reproducibility and reliability of the recorded spectra.

The specific surface area and textural properties of the biochar were determined *via* N_2_ adsorption–desorption isotherms evaluated at 77 K using a surface area analyzer (BELSORP MINI X). Prior to the measurement, approximately 0.07 g of the prepared material was outgassed under a vacuum at 150 °C for 8 h to thoroughly remove residual water and confined gases. The specific surface area was calculated using the Brunauer–Emmett–Teller (BET) method within the appropriate linear relative pressure range (*p*/*p*_0_ = 0.01–0.10). The pore size distribution was determined by applying the Non-Local Density Functional Theory (NLDFT) kernel appropriate for carbonaceous materials with slit-like pores.

### 2.4. Experimental design

Response Surface Methodology (RSM) using a Box–Behnken Design (BBD) was employed to optimize the adsorption parameters. The independent variables were adsorbent dosage (*X*_1_: 0.2–0.6 g L^−1^), initial pH (*X*_2_: 2–6), and initial SMX concentration (*X*_3_: 5–15 ppm).

### 2.5. Adsorption experiments

Batch adsorption experiments were conducted by adding a specific dosage of KA-BC-WFR to SMX solutions in Erlenmeyer flasks. The pH was adjusted using 0.1 M HCl or NaOH. The mixtures were agitated in a temperature-controlled shaker at 200 rpm. After the specified time, the solid was separated, and the residual SMX concentration was measured using a UV-Vis spectrophotometer at *λ*_max_ = 265 nm.

All absorbance data were measured three times and averaged to eliminate possible measurement errors. The adsorption equilibrium capacity *Q*_eq_ (mg g^−1^) and removal efficiency (% *R*) of SMX on the adsorbent were calculated *via* the following equations:1
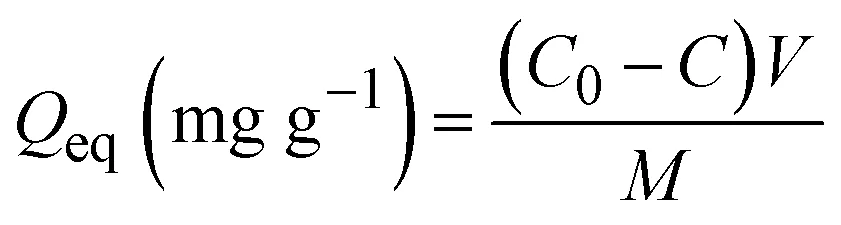
2
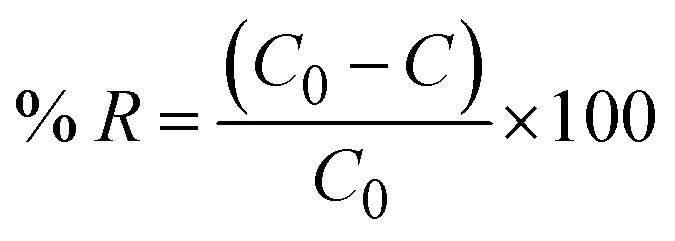
where *Q*_eq_ (mg g^−1^) is the amount of SMX adsorbed on the adsorbent, *C*_0_ (mg L^−1^) and *C* (mg L^−1^) are the initial concentration and the equilibrium concentration of SMX, respectively, *V* (L) is the volume of the solution, *M* (g) is the mass of the adsorbent used, and % *R* is the removal efficiency of the SMX.

The adsorption kinetics of the SMX/KA-BC-WFR system were calculated *via*eqn (3) for the pseudo-first-order kinetic model and eqn (4) for the pseudo-second-order kinetic model.

3 ln (*q*_e_ − *q*_*t*_) = ln *q*_e_ − *k*_1_*t*

4
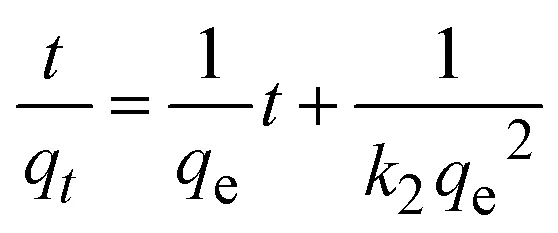
where *q*_e_ is the amount of adsorbed SMX per unit adsorbent at equilibrium (mg g^−1^), *q*_*t*_ is the amount of adsorbed SMX per unit adsorbent at any time (mg g^−1^), *t* the time (min), *k*_1_ is the rate of pseudo-1st order kinetic model (min^−1^).

## 3. Results and discussion

### 3.1. Characterization of adsorbent

#### 3.1.1. Surface morphology (SEM analysis) and textural properties

**Fig. 1:**
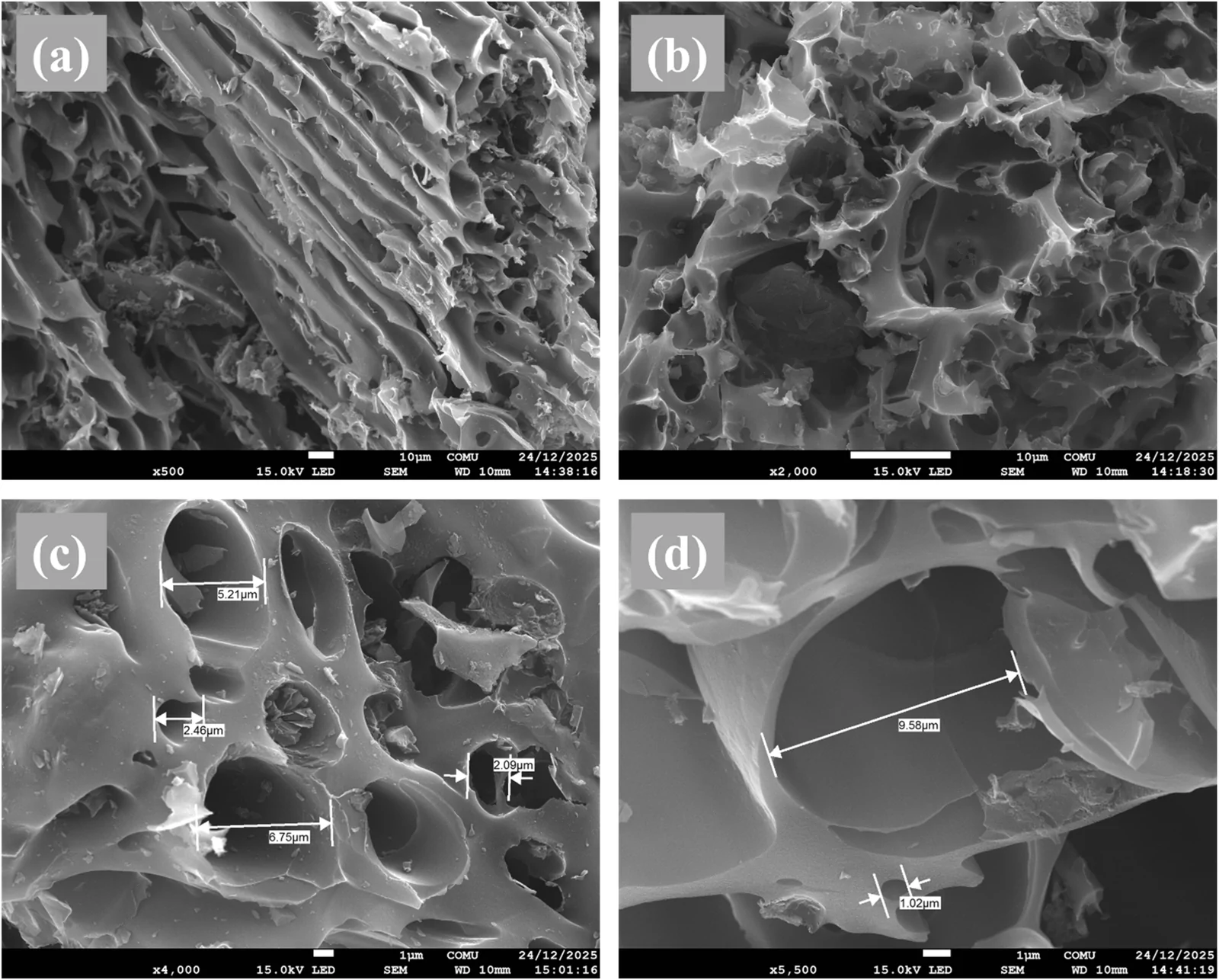
SEM images of KA-BC-WFR sample at different magnifications 500× (a), 2 000× (b), 4 000× (c), 5 500× (d).

The surface morphology and structural characteristics of the activated biochar were examined using Scanning Electron Microscopy (SEM). The micrographs presented in Fig. 1 reveal that the material possesses a highly developed, sponge-like porous structure. As shown in Fig. 1a and b, the surface is characterized by a network of interconnected cavities and irregular voids. This open, honeycomb-like architecture is critical for adsorption applications as it facilitates the diffusion of adsorbate molecules into the internal structure. Higher magnification images confirm the presence of macropores with a broad size distribution, indicating pore diameters ranging from approximately 1.0 µm to 9.6 µm.

**Fig. 2:**
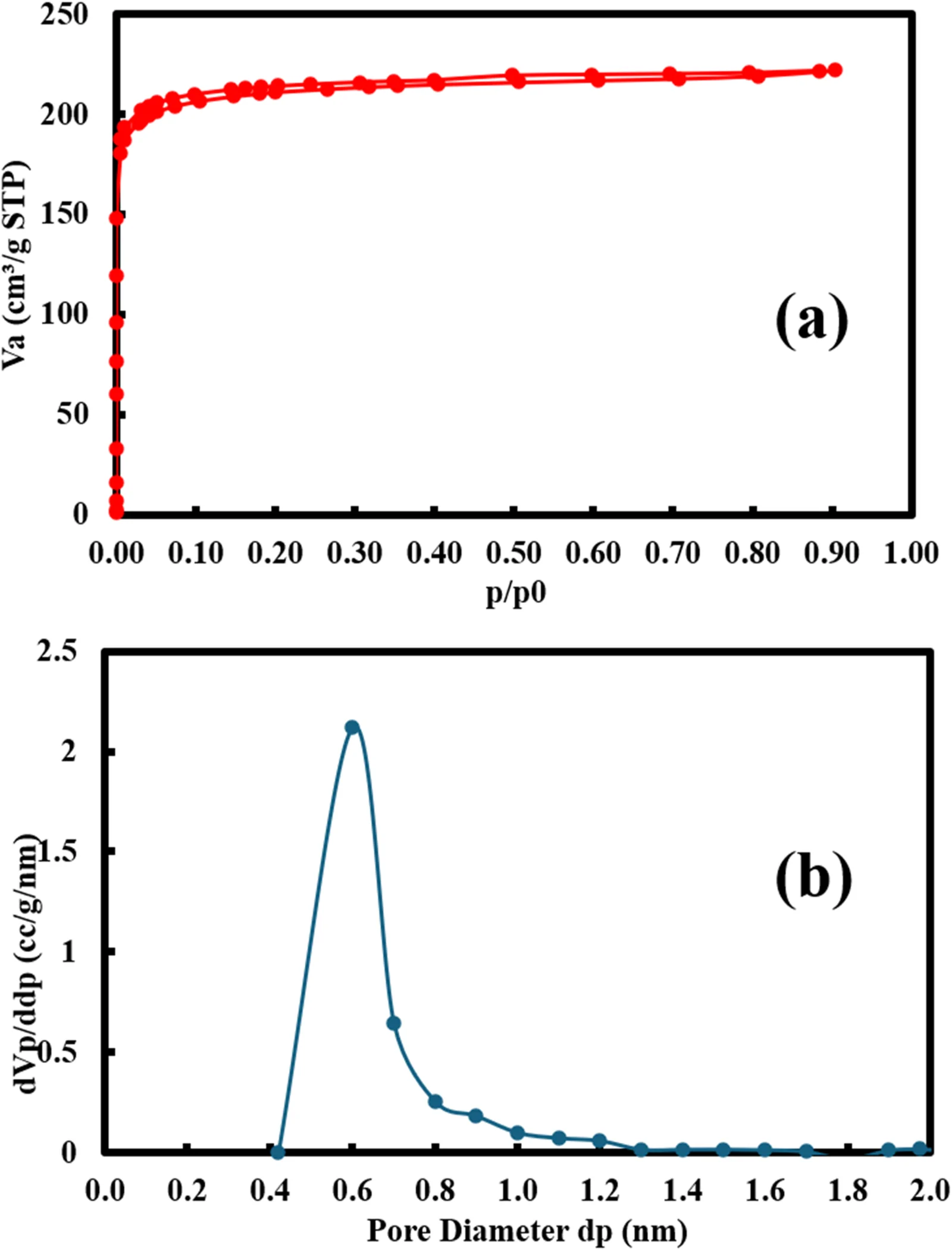
(a) N_2_ adsorption–desorption isotherm and (b) corresponding pore size distribution of KA-BC-WFR.

To further quantify the internal porosity, N_2_ adsorption–desorption analysis was conducted at 77 K. Fig. 2 shows the N_2_ adsorption–desorption isotherm and corresponding pore size distribution of KA-BC-WFR. The BET (Brunauer–Emmett–Teller) analysis revealed a remarkably high specific surface area of 828 m^2^ g^−1^. The total pore volume was determined to be 0.3465 cm^3^ g^−1^, with an average pore diameter of 1.30 nm. The modal pore diameter of 0.60 nm indicates that the internal surface area is predominantly microporous. The combination of SEM and BET results confirms that KA-BC-WFR possesses a hierarchical pore structure. The macropores observed in the SEM images (1–10 µm) act as low-resistance transport channels, allowing the bulky SMX molecules to rapidly access the dense network of internal micropores where the actual binding occurs.

#### 3.1.2. Point of zero charge

The surface charge behavior of KA-BC-WFR was evaluated using the pH drift method. A series of solutions with initial pH (pH_i_) values ranging from 2.08 to 10.53 were prepared. After the addition of the biochar and an equilibration period of 72 h, the final pH (pH_f_) values were measured. The pH_PZC_ is identified as the point where the change in pH (ΔpH = pH_f_ − pH_i_) equals zero. As detailed in Table 1, the intersection of the ΔpH *versus* pH_i_ curve with the *x*-axis indicates a pH_PZC_ of 6.24. This slightly acidic-to-neutral PZC is characteristic of well-washed, KOH-activated biochars and signifies that the adsorbent surface is net-positively charged at pH levels below 6.24 due to protonation, and net-negatively charged above this threshold.

**Table 1:** pH drift experimental data for the determination of pH_PZC_ of KA-BC-WFR

Initial pH (pH_i_)	Final pH (pH_f_)	ΔpH = pH_f_ − pH_i_
2.08	2.16	+0.08
4.13	4.90	+0.77
5.98	6.28	+0.30
8.11	6.02	−2.09
10.53	6.94	−3.59

#### 3.1.3. Surface functional groups (FTIR analysis)

**Fig. 3:**
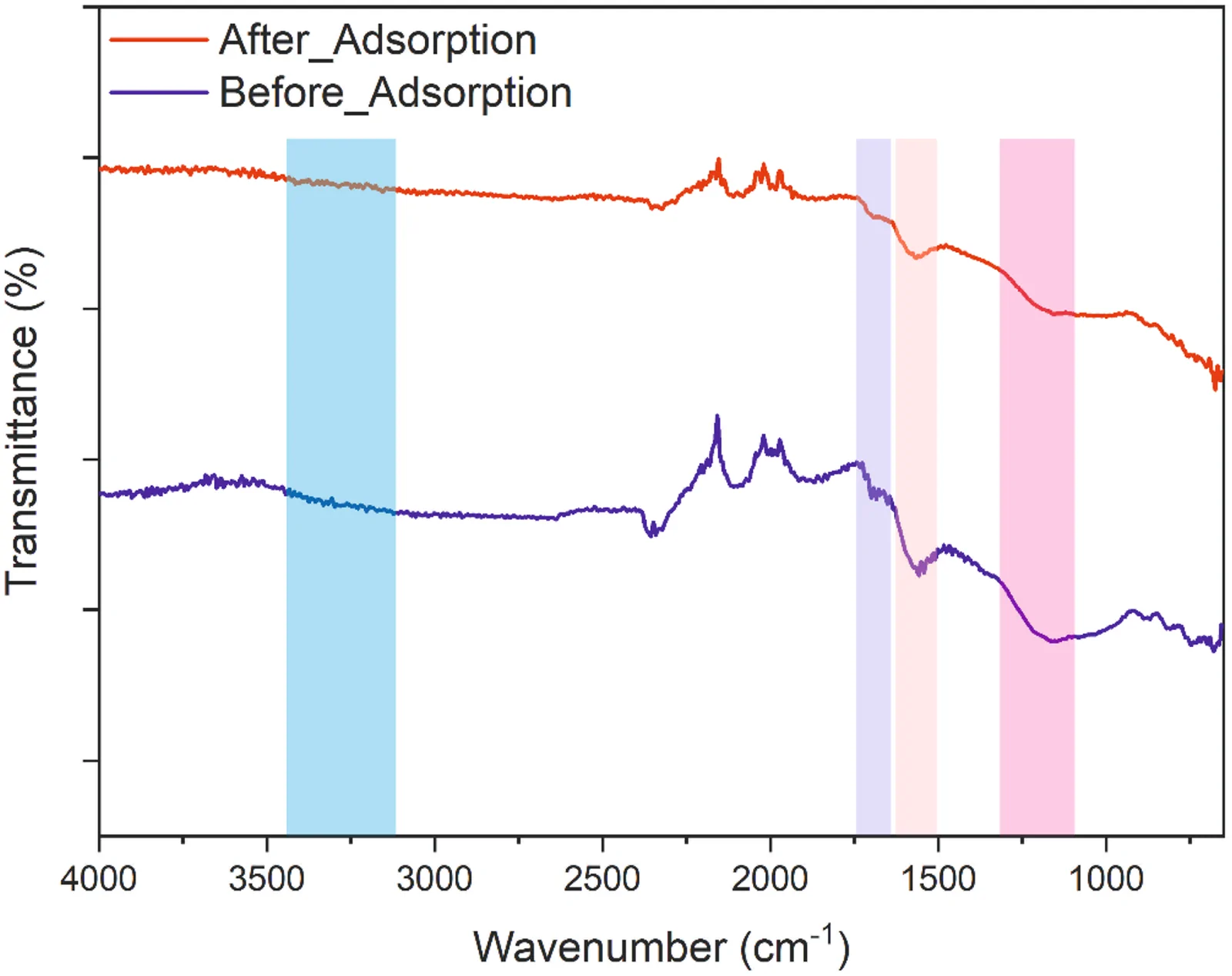
FTIR spectra of KA-BC-WFR before and after SMX adsorption.

To elucidate the specific functional groups responsible for the binding of sulfamethoxazole (SMX) and to verify the proposed adsorption mechanisms, Fourier Transform Infrared (FTIR) spectroscopy was conducted on the KA-BC-WFR biochar before and after adsorption (Fig. 3).

The spectrum of the pristine biochar (before_adsorption, blue line) reveals several characteristic peaks indicative of a highly functionalized carbonaceous surface resulting from the KOH activation. A broad absorption band observed in the region of 3400–3200 cm^−1^ (the band was labeled with blue color) is assigned to the stretching vibrations of hydroxyl (–OH) groups, which are associated with phenolic structures and adsorbed water molecules. The prominent peak located around 1580–1620 cm^−1^ (the bands were labeled with purple and orange) is attributed to the stretching vibrations of C=C bonds within the graphitic aromatic rings, overlapping with C=O stretching vibrations from oxygen-containing functional groups (such as carboxyls or ketones). Additionally, the broad band appearing between 1000 and 1200 cm^−1^ (the band was labeled with pink color) corresponds to C–O stretching vibrations (*e.g.*, from ether or ester groups).

Following the adsorption of SMX (after_adsorption, red line), noticeable alterations and shifts in the spectrum provide direct evidence of specific surface interactions. The broad –OH stretching band (∼3400 cm^−1^) exhibits a change in its profile and relative intensity. This modification strongly indicates the formation of hydrogen bonds between the oxygen-containing functional groups (hydroxyl/carboxyl) of the biochar acting as hydrogen bond donors/acceptors and the electronegative amine (–NH_2_) or sulfonyl (–SO_2_–) groups of the SMX molecules.

Furthermore, the aromatic C=C and C=O stretching band near 1600 cm^−1^ undergoes distinct modification and dampening after SMX loading. This alteration reflects the occurrence of strong π–π electron donor–acceptor interactions (stacking) between the electron-rich aromatic rings of the SMX molecules and the conjugated graphitic domains of the biochar framework. These spectral shifts substantiate the hypothesis that, alongside physical pore-filling, strong specific chemical interactions (hydrogen bonding and π–π stacking) are primary driving forces in the adsorption process, perfectly corroborating the pseudo-second-order kinetic data and the thermodynamically favorable nature of the system.

### 3.2. Optimization study *via* response surface methodology

#### 3.2.1. Model fitting and statistical analysis (ANOVA)

The optimization of sulfamethoxazole (SMX) adsorption onto KA-BC-WFR was carried out using a Box–Behnken Design (BBD). The interactive effects of three independent variables—adsorbent dosage (*X*_1_, 0.2–0.6 g L^−1^), initial pH (*X*_2_, 2.0–6.0), and initial SMX concentration (*X*_3_, 5–15 mg L^−1^)—on the SMX removal efficiency (%) were evaluated. A reduced second-order polynomial equation was fitted to the experimental data. The empirical relationship between the SMX removal efficiency (*Y*) and the independent variables in terms of coded factors is expressed by the following equation:

5 *Y* = 65.00 + 8.34*X*_1_ − 27.05*X*_2_ − 17.26*X*_3_ − 17.46*X*_2_*X*_3_ − 14.61*X*_1_^2^

The statistical significance and adequacy of the reduced quadratic model were rigorously evaluated using Analysis of Variance (ANOVA). As shown in Table 2, the overall model exhibited a high *F*-value of 19.48 and a statistically significant *p*-value of 0.0001 (*p* < 0.05), indicating that the regression model effectively captures the relationship between the operational parameters and the removal efficiency. The quality of the fit was supported by a high determination coefficient (*R*^2^) of 0.9154 and an adjusted *R*^2^ of 0.8684. Furthermore, the Adequate Precision value, which measures the signal-to-noise ratio, was 13.36. A ratio greater than 4 is desirable, confirming that this model provides an adequate signal and can be safely used to navigate the design space.

**Table 2:** Analysis of variance (ANOVA) for the quadratic response surface model

Source	Sum of squares	df	Mean square	*F*-Value	*p*-Value	Significance
Model	10 807.3	5	2161.46	19.48	0.0001	Significant
Adsorbent dose (*X*_1_)	555.94	1	555.94	5.01	0.052	
pH (*X*_2_)	5852	1	5852	52.74	<0.0001	
Initial conc. (*X*_3_)	2381.88	1	2381.88	21.47	0.0012	
*X*_2_ *X*_3_	1220.1	1	1220.1	11	0.009	
*X*_12_	797.37	1	797.37	7.19	0.0252	
Residual	998.6	9	110.96			
Lack of fit	989.17	7	141.31	29.97	0.0327	Significant
Pure error	9.43	2	4.72			
Cor. total	11 805.91	14				
***R*** ^**2**^ **= 0.9154**		**Adj-*R*** ^**2**^ **= 0.8684**		**Adeq precision = 13.36**		

An examination of the individual terms reveals that the linear main effects of pH (*p* < 0.0001) and initial SMX concentration (*p* = 0.0012) are highly significant. Additionally, the predictive capability of the model is strongly driven by the interactive effect between initial pH and SMX concentration (*X*_2_*X*_3_, *p* = 0.0090) and the quadratic effect of the adsorbent dosage (*X*_1_^2^, *p* = 0.0252).

#### 3.2.2. Interactive effects of operational parameters

**Fig. 4:**
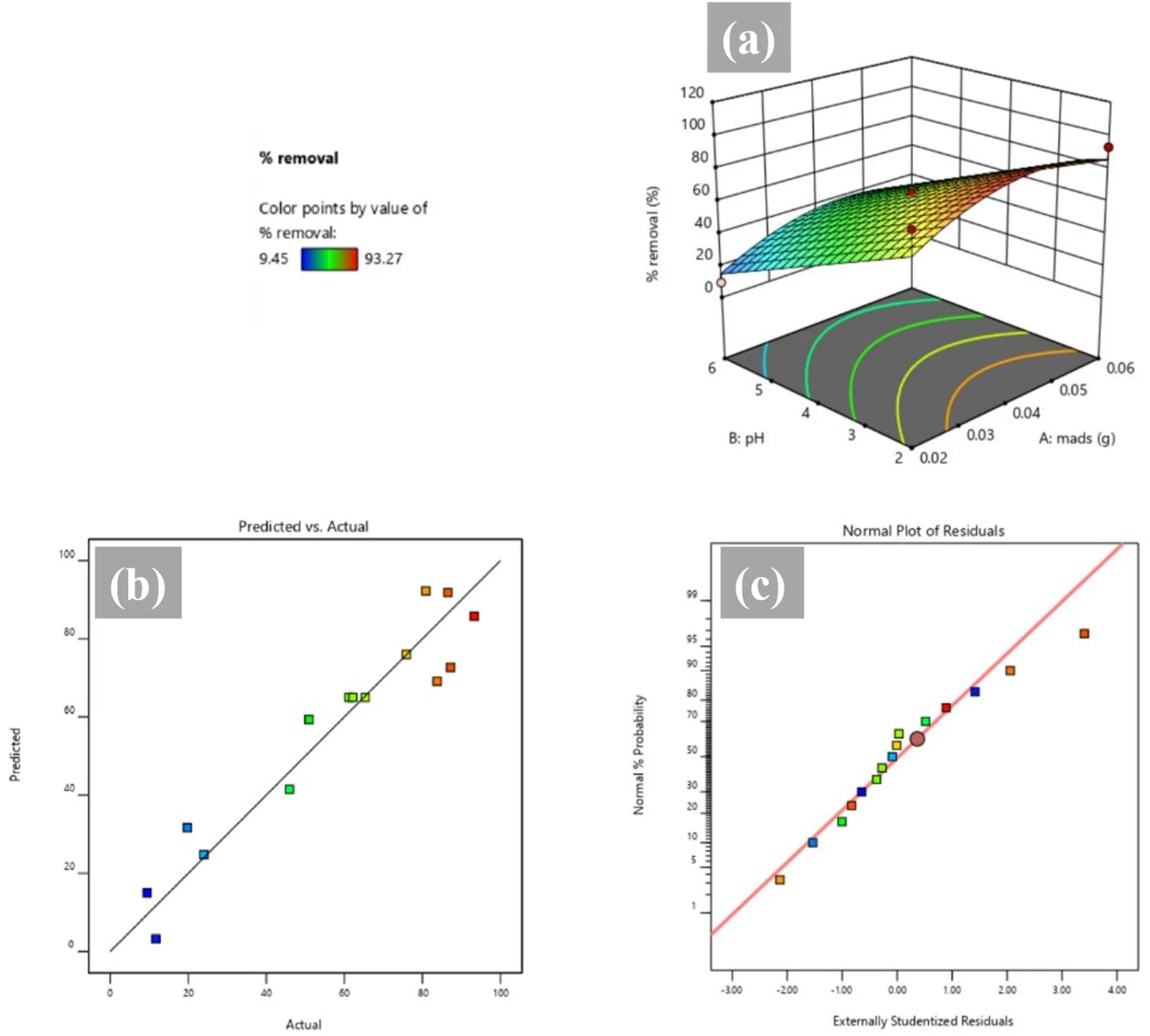
(a) The 3D surface plot, (b) predicted *vs.* actual plot, and (c) normal plot of residuals for % removal output.

The interactive and non-linear effects of the variables on SMX removal are visually represented through 3D response surface plots (Fig. 4). As indicated by the ANOVA results, the system is notably influenced by the interaction between pH and initial concentration, as well as the quadratic behavior of the adsorbent dosage.

This optimal adsorption at pH 2.0 is highly intriguing when correlated with our pH drift findings (pH_PZC_ = 6.24). At pH 2.0, the KA-BC-WFR surface is highly protonated and carries a strong positive charge. Concurrently, considering the dissociation constants of SMX (p*K*_a_1__ ≈ 1.6, p*K*_a_2__ ≈ 5.7), the SMX molecules are predominantly in their neutral zwitterionic or slightly cationic forms. Under these conditions, the exceptionally high removal efficiency demonstrates that the adsorption process is not reliant on electrostatic attraction. Instead, the strong specific chemical interactions (namely, powerful hydrogen bonding and π–π electron donor–acceptor interactions) coupled with the hierarchical pore-filling mechanism drive the adsorption process, successfully overcoming any potential repulsive barriers. Conversely, as the pH increases toward alkaline conditions (*e.g.*, pH > 6.24), both the biochar surface and the fully deprotonated SMX molecules become strongly negatively charged. This results in severe electrostatic repulsion, which explains the precipitous drop in removal efficiency to as low as 10%.

The robust predictive capability of the reduced quadratic model is visually confirmed by the tight correlation in the predicted *versus* actual diagnostic plot and the normal distribution of the residuals. Furthermore, the 3D response surface plot of pH *versus* adsorbent dosage illustrates the dominant antagonistic effect of increasing pH, alongside the optimal threshold plateau established by the quadratic dosage parameter. While maximizing the removal percentage was the primary optimization target, the corresponding adsorption capacity (*q*, mg g^−1^) was also evaluated to ensure efficient adsorbent utilization. An inherent trade-off was observed: higher adsorbent dosages provided more active sites and increased the overall removal percentage but resulted in a lower adsorption capacity per unit mass due to site unsaturation. This antagonistic relationship is mathematically confirmed by the negative coefficient for dosage (−5.09) in the reduced two-factor interaction (2FI) model developed for adsorption capacity (detailed ANOVA and regression equations for *q* are provided in the SI). By simultaneously evaluating both responses, the chosen optimal dosage (∼0.46 g L^−1^) represents a practical condition that maximizes pollutant removal from the water phase without causing excessive underutilization of the biochar.

#### 3.2.3. Optimization and experimental validation

The numerical optimization feature of the RSM methodology was utilized to identify the precise conditions for maximizing SMX removal. The mathematical model predicted a maximum theoretical removal efficiency of 94.78% under the optimal conditions of an adsorbent dosage of 0.46 g L^−1^, a pH of 2.0, and an initial SMX concentration of 5.0 mg L^−1^. These optimal parameters align perfectly with the experimental run exhibiting the highest measured removal (93.27% at 0.6 g L^−1^, pH 2.0, 10 mg L^−1^), confirming the reliability of the RSM model for optimizing the treatment of SMX-contaminated water using KA-BC-WFR.

### 3.3. Adsorption kinetics

The kinetic behavior of sulfamethoxazole (SMX) adsorption onto KA-BC-WFR was investigated to determine the equilibration time and elucidate the rate-controlling mechanism. The kinetic study was conducted under the conditions of an adsorbent amount (*m*_ads_) of 22 mg, solution volume (*V*) of 100 mL, pH = 3.0, initial SMX concentration (*C*_0,*i*_ = 10.76 mg L^−1^), and a constant temperature of 25 °C maintained *via* a water bath. The adsorption process exhibited a rapid initial uptake during the first 30 minutes, attributed to the abundance of vacant active sites on the adsorbent surface. Approximately 42% of the ultimate capacity was achieved within this initial phase. Following this, the adsorption rate gradually decreased as internal diffusion resistance increased and surface sites became saturated. The system approached equilibrium around 120–170 minutes, with a maximum experimental capacity (*q*_e,exp_) of 29.34 mg g^−1^.

**Fig. 5:**
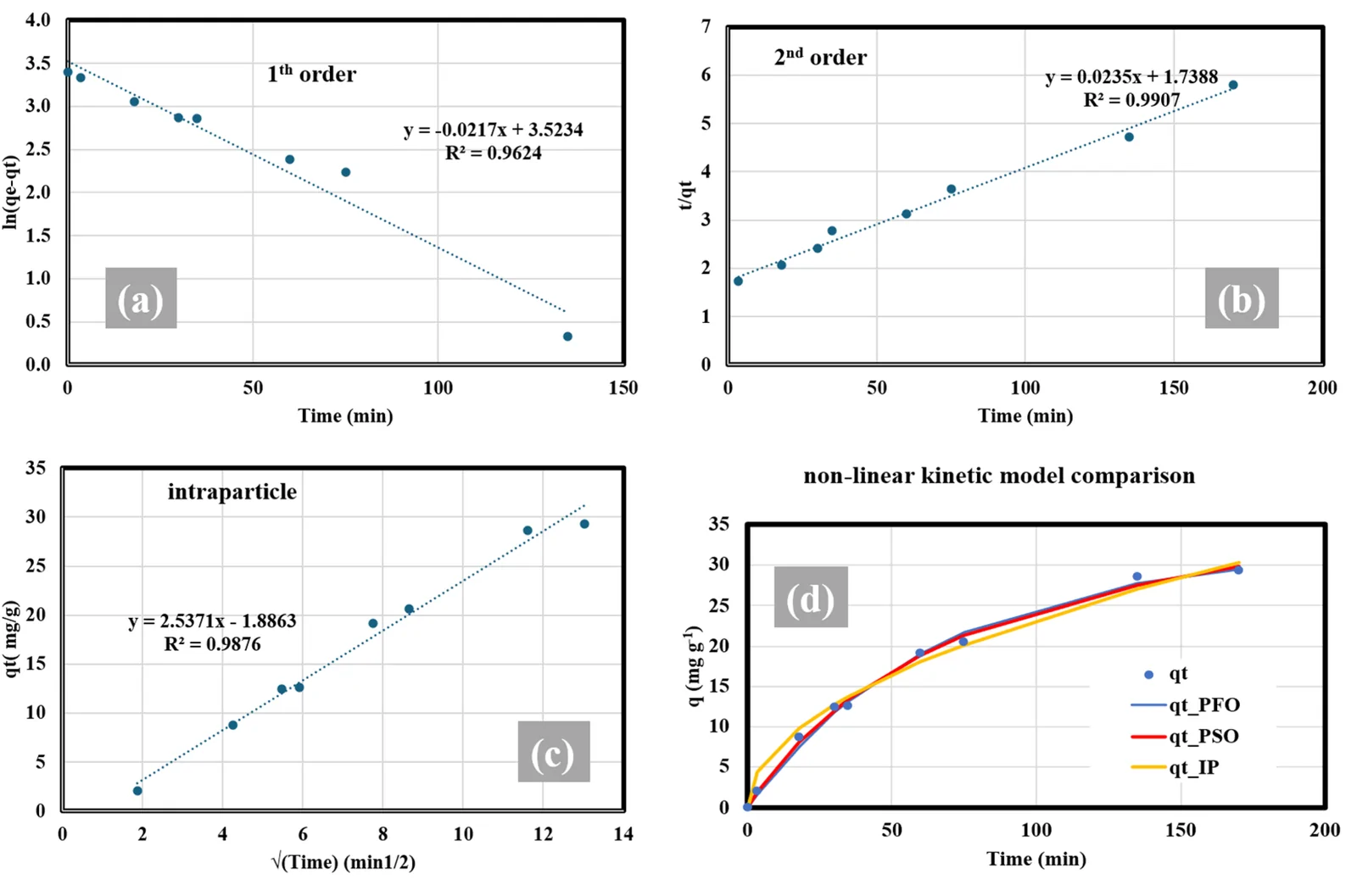
Analysis of the adsorption kinetics. (a) Linearized pseudo-first-order (PFO) model, (b) linearized pseudo-second-order (PSO) model, (c) intraparticle diffusion (Weber–Morris) model, (d) nonlinear kinetic model comparison.

To avoid the error structure distortion inherent in linearizing kinetic equations, the data were evaluated using non-linear regression for the pseudo-first-order (PFO) and pseudo-second-order (PSO) models. The validity of the fits was compared using the Root Mean Square Error (RMSE) and Mean Absolute Error (MAE). As detailed in Table 3 and Fig. 5, the PSO model exhibited lower error metrics (RMSE = 0.62 mg g^−1^, MAE = 0.56 mg g^−1^) compared to the PFO model (RMSE = 0.71 mg g^−1^, MAE = 0.61 mg g^−1^), indicating a superior fit to the experimental data. While the calculated theoretical equilibrium capacity from the PSO model (*q*_e,cal_ = 43.72 mg g^−1^) overestimates the experimental capacity (*q*_e,exp_ = 29.34 mg g^−1^), this is a recognized phenomenon in batch systems where strong physicochemical interactions slowly reach absolute equilibrium. The better fit of the PSO model suggests that the overall rate-limiting step is predominantly influenced by chemisorption. This mechanism involves strong specific interactions, including hydrogen bonding and π–π stacking between the SMX molecules and the surface functional groups of KA-BC-WFR, which is highly consistent with our FTIR and thermodynamic findings.

**Table 3:** Kinetic parameters for the adsorption of SMX onto KA-BC-WFR

Model	Parameter	Value	Unit
Experimental	*q*_e,exp_	29.34	mg g^−1^
Pseudo-first order	*k*_1_	0.0153	min^−1^
	*q*_e,cal_	31.88	mg g^−1^
	**RMSE**	**0.71**	mg g^−1^
	**MAE**	**0.61**	mg g^−1^
Pseudo-second order	*k*_2_	2.9 × 10^−4^	g mg^−1^ min^−1^
	*q*_e,cal_	43.72	mg g^−1^
	**RMSE**	**0.62**	mg g^−1^
	**MAE**	**0.56**	mg g^−1^
Intraparticle diffusion (Weber–Morris)	*k*_int_	2.452	mg g^−1^ min^−1/2^
	*C*	−1.127	mg g^−1^
	***R*** ^**2**^	**0.9876**	—

Furthermore, the intraparticle diffusion model (Weber–Morris) was applied to elucidate the mass transfer mechanisms. The linear plot of *q*_*t*_*versus t*^1/2^ did not pass through the origin (*C* = −1.127 ≠ 0), indicating that intraparticle diffusion is not the sole rate-controlling step; rather, a multi-step mechanism governs the adsorption process. The negative intercept (*C*) value reveals a significant boundary layer effect and implies that external mass transfer (film diffusion) acts as a primary resistance during the initial stages of adsorption, before the SMX molecules rapidly diffuse into the hierarchical macroporous transport channels observed in the SEM analysis.

### 3.4. Adsorption isotherms

**Fig. 6:**
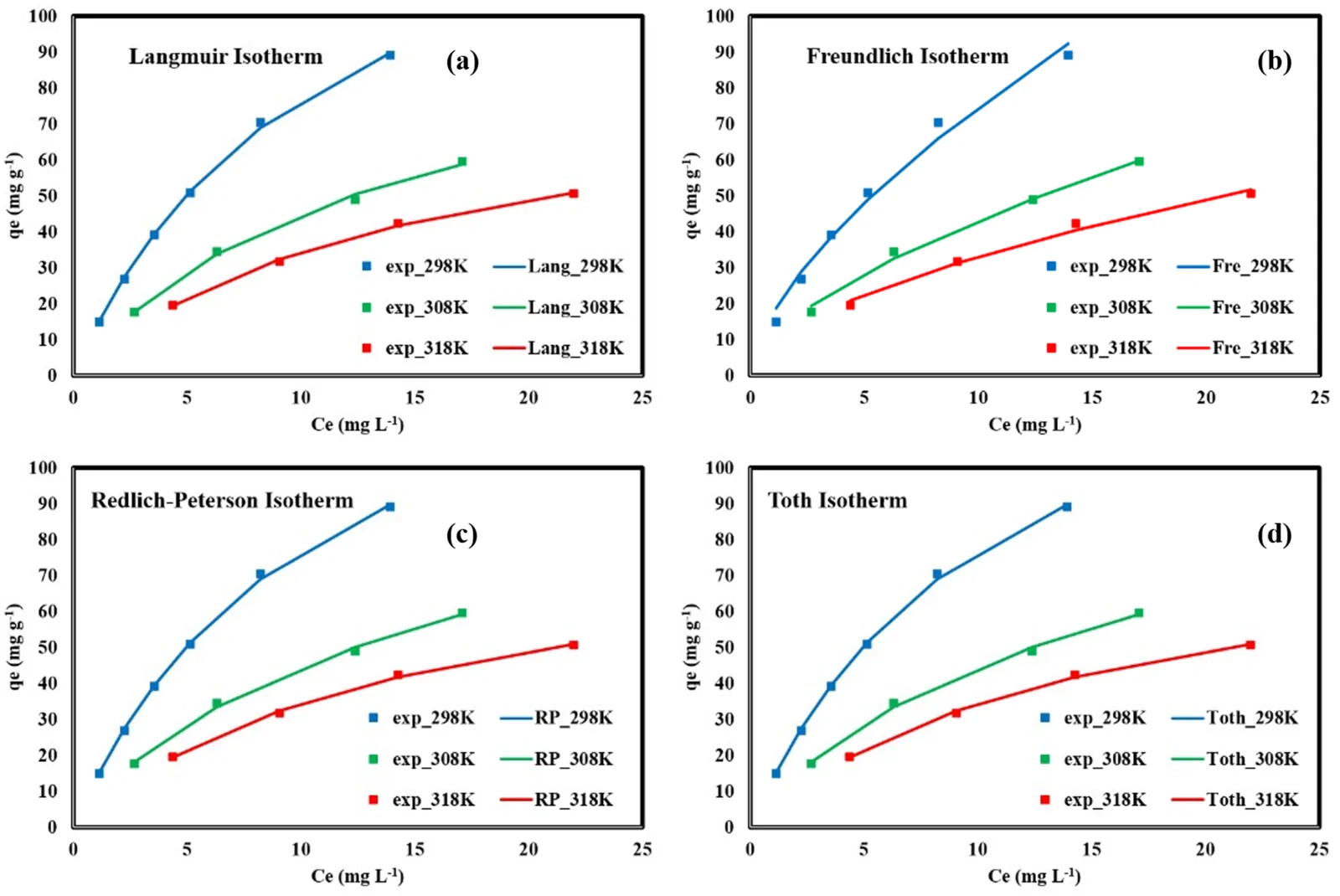
Adsorption isotherm fittings with (a) Langmuir, (b) Freundlich, (c) Redlich–Peterson, (d) Toth isotherm models.

The equilibrium adsorption isotherms of SMX were studied at 298, 308, and 318 K. Four different adsorption isotherm models were tested to fit the experimental data. Fig. 6 shows the fittings of the four adsorption isotherm models (Langmuir, Freundlich, Redlich–Peterson, and Toth) with respect to experimental data. The experimental data were best described by the three-parameter models, specifically the Redlich–Peterson and Toth isotherms, which consistently yielded the highest correlation coefficients (*R*^2^ > 0.998 at 298 and 318 K). The superior fit of these models suggests that the adsorbent surface is not entirely homogeneous but rather possesses a heterogeneous distribution of active sites.

Among the two-parameter models, the Langmuir model provided a satisfactory fit (*R*^2^ > 0.99), suggesting significant monolayer coverage. The maximum monolayer adsorption capacity (*q*_max_) calculated from the Langmuir model was found to be 160.05 mg g^−1^at 298 K. As the temperature increased from 298 K to 318 K, the adsorption capacity and Langmuir constants decreased, consistent with the exothermic nature of the process. Table 4 shows the isotherm model equations and parameters for SMX adsorption at different temperatures. Table 5 summaries the maximum adsorption capacity of SMX onto adsorbent in this study and in the literature. According to the comparison table, KOH-activated biochar (KA-BC-WFR) is favorable alternative for SMX removal from water media.

**Table 4:** Isotherm model parameters for SMX adsorption at different temperatures

Model	Parameter	298 K	308 K	318 K
Langmuir	*q*_max_ (mg g^−1^)	160.05	103.44	84.79
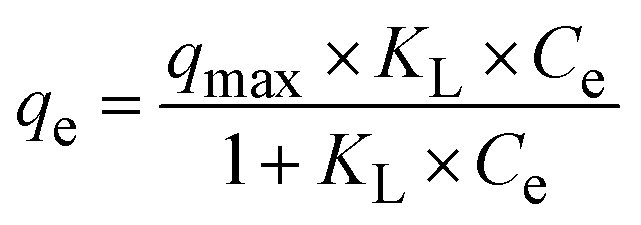	*K*_L_ (L mg^−1^)	0.091	0.076	0.067
	***R*** ^**2**^	**0.9974**	**0.9915**	**0.9981**
Freundlich	*K*_F_	17.25	10.56	9.06
*q*_e_ = *K*_F_ × *C*_e_^(1/*n*)^	*n*	1.57	1.63	1.77
	***R*** ^**2**^	**0.9882**	**0.9903**	**0.9919**
Redlich–Peterson	*K*_RP_ (L mg^−1^)	14.69	9.47	5.75
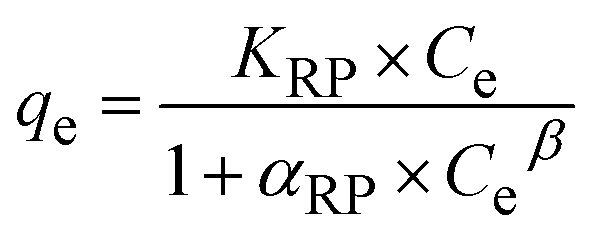	*α*_RP_	0.091	0.179	0.067
	*β*_RP_	1.00	0.79	1.00
	***R*** ^**2**^	**0.9991**	**0.9969**	**0.9988**
Toth	*q*_max_ (mg g^−1^)	160.05	155.97	84.79
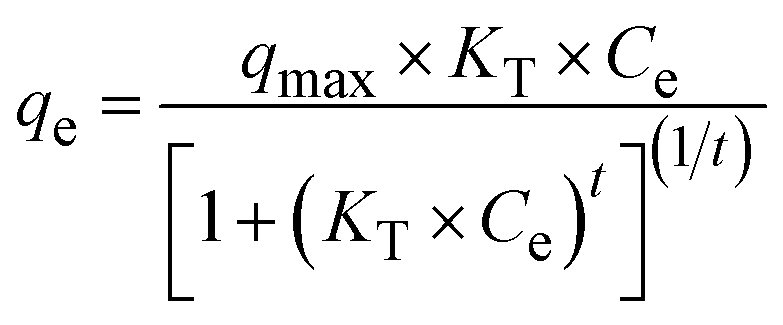	*K*_Toth_ (L mg^−1^)	0.091	0.063	0.067
	*t*	1.00	0.68	1.00
	***R*** ^**2**^	**0.9991**	**0.9967**	**0.9988**

**Table 5:** Comparison table SMX adsorption capacities

Adsorbent	Adsorption capacity (mg g^−1^)	Experimental conditions	Reference
KOH-activated BC-wildfire residue (KA-BC-WFR)	160.05	pH = 3, 25 °C, 3 h shaking, *C*_0_ = 10 mg L^−1^	This study
Powdered activated carbon (Pinus tree)	130.73	pH = 7, 25 °C, 2 h contact	17
KOH-activated corn stover biochar	76.92	pH = 7, 25 °C, 24 h shaking	18
H_3_PO_4_-activated corn stover biochar	70.42		
Municipal sludge-derived biochar (SBC)	11.56	pH = 7, 20 °C, 24 h shaking	19
Amine grafted activated carbon	53.47	pH = 6, 15 °C, 1 h shaking	20
Graphene-like carbon	289.23	pH = 6, 20 °C, 1 h shaking	21
AC-reed canary grass	160.5	pH = 4, 25 °C, 24 h shaking	22
Bermudagrass-derived biochar	425	pH = 3, 25 °C, 1 h shaking, *C*_0_ = 100 mg L^−1^	23
Activated BC	88.10	pH = 3, 25 °C, 24 h shaking, *C*_0_ = 100 mg L^−1^	24
Alfalfa-derived BC	88	pH = 5, 25 °C, 120 h shaking, *C*_0_ = 100 mg L^−1^	25

### 3.5. Adsorption thermodynamics

The thermodynamic parameters such as Gibbs free energy change (Δ*G*°), enthalpy change (Δ*H*°), and entropy change (Δ*S*°), were calculated using the Van't Hoff equation and are shown in Table 6. To ensure dimensional consistency, the thermodynamic equilibrium constant (*K*_eq_) must be dimensionless. Therefore, *K*_eq_ was derived from the Langmuir isotherm constant (*K*_L_, L mg^−1^) by multiplying it by the molecular weight of SMX (*M*_w_ = 253.28 g mol^−1^) and a unit conversion factor (1000 mg g^−1^), and subsequently dividing by the standard state concentration (*C*_0_ = 1 mol L^−1^), as shown in the following equation:6
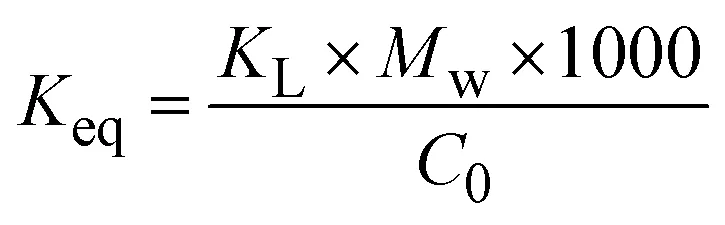


**Table 6:** Thermodynamic parameters for SMX adsorption

Temp. (K)	*K*_eq_	Δ*G*° (kJ mol^−1^)	Δ*H*° (kJ mol^−1^)	Δ*S*° (J mol^−1^ K^−1^)
298	23 260.68	−24.91	**−11.93**	**+43.48**
308	19 471.84	−25.29		
318	17 189.35	−25.78		

Using this approach at 298 K (*K*_L_ = 0.0918 L mg^−1^), the dimensionless *K*_eq_ was calculated to be 23 260.28. The negative values of Δ*G*° obtained at all temperatures (ranging from −24.91 to −25.78 kJ mol^−1^) indicate that the adsorption is spontaneous and thermodynamically favorable.

The standard enthalpy change (Δ*H*°) was calculated to be −11.93 kJ mol^−1^. The negative value confirms the exothermic nature of the adsorption process. This magnitude is characteristic of strong physical adsorption enhanced by hydrogen bonding, rather than strong covalent bonding. The positive value of Δ*S*° (+43.48 J mol^−1^ K^−1^) reflects an increase in randomness at the solid-solution interface, likely attributed to the displacement of water molecules from the hydration shells of the SMX species.

### 3.6. Proposed adsorption mechanism

Based on the kinetic, thermodynamic, and characterization data, the adsorption of SMX onto KA-BC-WFR is governed by a synergistic mechanism involving three primary interactions:

#### 3.6.1. Pore filling and diffusion

The N_2_ adsorption–desorption analysis (Type I isotherm) and the determined modal pore width of 0.60 nm and average pore diameter of 1.30 nm indicate that the material is dominated by ultramicropores. The actual adsorption and “pore filling” of the SMX molecules occur within this dense microporous network. Concurrently, the 1.0–9.6 µm structural openings observed in the SEM micrographs act as macroscopic transport channels, minimizing boundary layer resistance and allowing the SMX molecules to rapidly diffuse into the internal ultramicropores where the binding takes place.

#### 3.6.2. Hydrogen bonding and electrostatic interactions

At the optimal condition of pH 2.0, the biochar surface is net-positively charged (pH < pH_PZC_ of 6.24). Considering the dissociation constants of SMX (p*K*_a_1__ ≈ 1.6, p*K*_a_2__ ≈ 5.7), the SMX molecules exist predominantly in their neutral form at pH 2.0, alongside a minor cationic fraction. Because the majority of SMX is neutral, electrostatic interactions are approximately zero and do not act as a significant barrier. Specifically, the functional groups on the adsorbent surface act as strong hydrogen bond donors and acceptors. The prominent FTIR peak shifts confirm that the electronegative amine and sulfonyl groups of the SMX molecules form robust hydrogen bonds with the oxygen-containing functional groups (such as hydroxyls) of the biochar.

#### 3.6.3. π–π Stacking

The aromatic rings present in the SMX structure engage in strong π–π electron donor–acceptor (stacking) interactions with the conjugated graphitic domains of the biochar framework. This is supported by the distinct modification and dampening of the aromatic C=C and C=O stretching band near 1600 cm^−1^ in the FTIR spectrum following SMX loading.

#### 3.6.4. Thermodynamic and isotherm alignment

The magnitude of the calculated standard enthalpy change (Δ*H*° = −11.93 kJ mol^−1^) perfectly corroborates this proposed mechanism. This energy value falls within the characteristic range for strong physisorption and moderate-energy specific interactions (such as hydrogen bonding and π–π stacking), confirming that the process is not driven by irreversible covalent bond formation. Furthermore, the strong adherence of the equilibrium data to the Langmuir model among the two-parameter isotherms indicates that these specific hydrogen bonding and π–π stacking interactions coordinate the SMX molecules into a highly organized, single monolayer across the finite active sites of the biochar, preventing multi-layer accumulation.

## 4. Conclusion

In this study, a novel KOH-activated biochar derived from *Pinus brutia* wildfire residues (KA-BC-WFR) was successfully synthesized and applied for the efficient removal of sulfamethoxazole (SMX) from aqueous solutions. Structural and textural characterization revealed a hierarchical pore architecture; the biochar exhibited an exceptionally high BET specific surface area of 828 m^2^ g^−1^ dominated by micropores (average diameter 1.30 nm), complemented by a macroporous sponge-like morphology (1.0 to 9.6 µm) that facilitated rapid mass transfer and diffusion of the adsorbate. The point of zero charge (pH_PZC_) was determined to be 6.24 *via* the pH drift method, highlighting that strong specific chemical interactions overcome electrostatic repulsions under highly acidic conditions.

The adsorption process was statistically optimized using a Box–Behnken Design (RSM), identifying highly acidic conditions (pH 2.0) and a dosage of ∼0.46 g L^−1^ as the optimal parameters for maximum SMX removal.

Kinetic modeling revealed that the adsorption rapidly approached equilibrium within 120 minutes and perfectly aligned with the pseudo-second-order model (*R*^2^ = 0.9907), underlining chemisorption as the overall rate-limiting step. Furthermore, intraparticle diffusion analysis indicated a multi-step mass transfer mechanism where external film diffusion acts as a significant initial boundary layer resistance, evidenced by a negative intercept. Equilibrium data were well-described by non-linear isotherm models, with the Langmuir maximum monolayer capacity determined as 160.05 mg g^−1^ at 298 K. Rigorous thermodynamic analysis validated the spontaneous (Δ*G*° < 0) and exothermic (Δ*H*° = −11.93 kJ mol^−1^) nature of the process, while the positive entropy (Δ*S*° = +43.49 J mol^−1^ K^−1^) highlighted the displacement of water molecules from the hydration shells during adsorption. Collectively, the synergistic mechanism of hierarchical pore filling, strong hydrogen bonding, and π–π stacking confirms that KA-BC-WFR is a highly efficient and thermodynamically stable adsorbent for the sustainable remediation of pharmaceutical contaminants. Overall, the results substantiate that converting wildfire residues into activated biochar represents a highly effective, sustainable, and kinetically fast strategy for the remediation of pharmaceutical contaminants in wastewater treatment applications.

## Author contributions

The author solely conceived and designed the study, performed the data analysis, interpreted the results, prepared the original draft, critically revised the manuscript, and approved the final version for publication. The author accepts responsibility for all aspects of the work and its scientific integrity.

## Conflicts of interest

The author declares no conflict of interest.

## Data Availability

All data underlying the results presented in this study are available within the manuscript. Raw datasets obtained during the experimental and modeling phases are available from the corresponding author upon reasonable request.
